# Trends in antipsychotic prescribing among community-dwelling older adults with dementia, 2010-2018

**DOI:** 10.1093/haschl/qxaf021

**Published:** 2025-02-26

**Authors:** Annie W Yang, Mei Leng, Julia Cave Arbanas, Chi-Hong Tseng, A Mark Fendrick, Catherine Sarkisian, Cheryl L Damberg, Nina T Harawa, John N Mafi

**Affiliations:** Division of General Internal Medicine and Health Services Research, David Geffen School of Medicine at UCLA, Los Angeles, CA 90024, USA; Division of General Internal Medicine and Health Services Research, David Geffen School of Medicine at UCLA, Los Angeles, CA 90024, USA; Division of General Internal Medicine and Health Services Research, David Geffen School of Medicine at UCLA, Los Angeles, CA 90024, USA; Division of General Internal Medicine and Health Services Research, David Geffen School of Medicine at UCLA, Los Angeles, CA 90024, USA; Internal Medicine, Health Management and Policy, University of Michigan, Ann Arbor, MI 48109, USA; Division of General Internal Medicine and Health Services Research, David Geffen School of Medicine at UCLA, Los Angeles, CA 90024, USA; Greater Los Angeles Healthcare System Geriatrics Research Education and Clinical Center (GRECC), Los Angeles, CA 90073, USA; RAND Health, RAND Corporation, Santa Monica, CA 90401, USA; Division of General Internal Medicine and Health Services Research, David Geffen School of Medicine at UCLA, Los Angeles, CA 90024, USA; Division of General Internal Medicine and Health Services Research, David Geffen School of Medicine at UCLA, Los Angeles, CA 90024, USA; RAND Health, RAND Corporation, Santa Monica, CA 90401, USA

**Keywords:** low-value care, dementia, antipsychotics, older adults, quality of care

## Abstract

Due to an FDA “black box” warning for heightened risk of death, Choosing Wisely (CW) recommends avoiding antipsychotic prescription drugs as first-line treatment for dementia-related agitation. Yet, post-CW trends among community-dwelling patients with dementia remain unknown. In this retrospective cohort study, we used nationally representative Health and Retirement Study survey data linked to Medicare fee-for-service claims (January 1, 2010-December 31, 2018) to analyze prescribing trends during the pre-publication (2010-2012), publication (2013-2015), and post-publication (2016-2018) periods of CW recommendations. We included community-dwelling adults aged ≥65 years with dementia. We utilized multivariable mixed regression models to determine the percentage of patients prescribed any, potentially low-value, and potentially indicated antipsychotics. Among an estimated 2.4-2.7 million patients with dementia, any antipsychotic prescribing increased from 9.4% (95% CI, 6.4%-12.3%) during the pre-publication period (2010-2012) to 15.8% (95% CI, 12.8%-18.8%) (*P* < 0.001) during the publication period (2013-2015). Potentially low-value and potentially indicated prescriptions also increased. Post-publication period (2016-2018) prescribing of 16.0% (95% CI, 13.0%-19.1%) (*P* < 0.001) remained higher than pre-publication. Among older Americans with dementia, antipsychotic prescriptions increased after the publication of CW recommendations and held steady in the subsequent post-publication period. Stronger interventions, such as electronic clinical decision support tools and financial incentives, are needed to curb low-value antipsychotic prescribing for this vulnerable population.

## Teaser

Antipsychotic drugs have a “black box” warning for a heightened risk of death if used to treat behavioral agitation in patients with dementia. Multiple medical societies recommend against using antipsychotics in this way. Prescriptions among institutionalized older adults with dementia have declined over the past decade, but prescribing trends in community-dwelling patients with dementia in relation to medical society recommendations remain unknown. Our study shows that antipsychotic prescriptions among community-dwelling older Americans with dementia increased during the publication of multiple Choosing Wisely society recommendations and did not decline after their publication. Stronger interventions are needed to curb overprescribing for this vulnerable group.

## Introduction

The hazards of prescribing antipsychotic drugs have been well-documented among patients with dementia, a population expected to reach 11.6 million within the United States by 2040.^1^ Its numerous harmful adverse effects include an increased risk for falls and stroke.^2^ A 2005 meta-analysis reported heightened mortality risk for patients with dementia receiving antipsychotics, promptly leading to an FDA “black box” warning.^2^ Despite this knowledge, prescribing persisted among institutionalized older adults, resulting in a series of investigations and stringent federal oversight within nursing homes.^3,4^

By 2011, these investigations culminated in CMS implementing a policy to penalize nursing homes for overprescribing these drugs.^4^ Concurrently, the Choosing Wisely (CW) campaign launched in 2012, an international campaign intended to raise awareness among clinicians and patients regarding low-value care. CW involved over 80 specialty societies, 30 countries, and over 700 recommendations released to lay public through media coverage and Consumer Reports. Its specialty society partners included the American Geriatrics Society (AGS), the Society for Post-Acute and Long-Term Care Medicine (AMDA), and the American Psychiatric Association (APA), who all published strong recommendations between 2013 and 2015 discouraging first-line use of antipsychotics for dementia-related agitation.^5,6^ Subsequently, studies have documented declines in antipsychotic prescriptions among institutionalized older adults with dementia.^4^ To our knowledge, however, CMS has not monitored antipsychotic prescriptions among community-dwelling adults with dementia, and few studies have analyzed recent antipsychotic prescribing trends in this population.^7^

To examine prescribing trends of antipsychotics in relation to 3 specialty societies releasing CW recommendations discouraging antipsychotic use for treating dementia-related behavioral symptoms published between 2013 and 2015, we used nationally representative survey data from the Health and Retirement Study (HRS) linked to Medicare fee-for-service claims. We hypothesized that prescribing of this drug class with important toxicities would decline over time among community-dwelling adults, similar to prescribing patterns in institutionalized populations.

## Data and methods

The HRS is a nationally representative, biennial survey administered to US adults > 50 years old. We analyzed HRS-Medicare linked claims data and used previously validated cognitive measures^8-10^ to identify community-dwelling, Medicare fee-for-service beneficiaries ≥ 65 years old with dementia and ≥3 years of continuous Medicare parts A, B, and D coverage for each year between 2010 and 2018 (Supplement).

The percentage of patients prescribed any antipsychotic in each year was identified using part D prescription drug claims and National Drug Codes (NDC) codes (Table S1). Prescriptions were categorized into 4 categories: (1) all, (2) potentially indicated (eg, hospice and schizophrenia), (3) possibly low-value (eg, moderate depression), and (4) probably low-value (no coded indications) (Table S2). Due to rare instances of possibly low-value prescriptions, possibly and probably low-value prescriptions were combined into the single measure: “potentially low-value.”

We stratified prescription patterns by patient race/ethnicity as well as by socioeconomic status, using 2 proxy measures for socioeconomic disadvantage per Medicare: part D premium low-income subsidy (LIS) qualification or Medicare-Medicaid dual eligibility, combined into a single category hereafter known as “LIS-dual.”

We compared antipsychotic prescribing rates across 3 time periods: 2010-2012 (pre-publication period), 2013-2015 (publication of recommendations by AGS, AMDA, and APA), and 2016-2018 (post-publication period). Our multivariable mixed regression models estimated prescribing rates after adjusting for age, sex, and chronic comorbidities using the Wei multimorbidity index.^11^ For the primary outcome of “all prescriptions,” we applied the Bonferroni correction to control the type I error rate at the 5% level, to address multiple testing between 3 pair-wise timeframe comparisons. We accounted for survey stratification and clustering and applied survey weights for national representativeness and response rate. For further methodological details, see the Supplement.

All data were analyzed using SAS, version 9.4 (SAS Institute). The UCLA IRB approved this study, and we followed the Strengthening the Reporting of Observational Studies in Epidemiology guidelines (Table S3).

## Results

Our final sample of 858 unique participants represented 6.1% of all HRS-Medicare participants ≥ 65 years old (Figure S1, CONSORT diagram). Across the 3 timeframes, the sample ranged from 811-1080 patient-years, representing approximately 2.4-2.7 million community-dwelling, Medicare beneficiaries living with dementia (Table 1). The unweighted samples shown in Table 1 could be greater than the number of unique participants (*N* = 858) because a beneficiary may appear more than once within a given timeframe.

**Table 1.** Demographics of all Medicare fee-for-service beneficiaries ≥ 65 years old living with dementia.

	2010-2012	2013-2015	2016-2018
Unweighted sample, total person-years	1080	928	811
Weighted population estimate, millions	2.7	2.4	2.6
Weighted percentages (%)^a^			
Age			
65-75	19	20	19
75-84	35	35	39
85+	45	45	42
Sex			
Male	31	35	32
Female	69	65	68
Race/ethnicity			
NH-White	60	62	63
NH-Black	18	16	16
Hispanic	18	19	18
Other	4	3	3
MWI, mean (SD)^b^	10.6 (7.1) Range 0-46	10.6 (6.8) Range 0-46	11.3 (6.9) Range 0-40
LIS-dual			
Yes	69	60	53
No	31	40	47
Dementia severity			
Mild	42	39	40
Moderate	22	24	16
Severe	36	37	44
Caregiver present			
Yes	69	62	66
No	31	38	34
Geographic location^c^			
Northeast	16	16	13
Midwest	17	20	22
South	55	53	49
West	12	11	16
Education, years			
<12	63	63	46
12	22	22	25
13-15	9	8	17
>16	6	8	12
Respondent			
Self	63	69	67
Proxy	37	31	33

Most were self-respondents, female, non-Hispanic White, lived in the Southern United States, and had a caregiver. ^a^May sum to >100% due to rounding. ^b^The MWI descriptive statistics were calculated for the unweighted sample. ^c^“Other” geographic location was not reported due to cell sizes < 10 and thus may not sum to the total. Abbreviations: LIS-dual, qualifying for either a part D premium low-income subsidy (LIS) or being dual-eligible for both Medicare and Medicaid; MWI, multimorbidity weighted index; NH, non-Hispanic; SD, standard deviation.

The population-weighted prescribing rates were 121 (95% CI, 93-148), 142 (95% CI, 99-185), and 142 (95% CI, 107-177) per 1000 patients (Figure S2) across the 3 timeframes. The adjusted percentage of patients prescribed antipsychotics increased from 9.4% (95% CI, 6.4%-12.3%) during the pre-publication period (2010-2012) to 15.8% (12.8%-18.8%; *P* < 0.001) during the publication period (2013-2015). Compared with the pre-publication period, prescriptions during the post-publication period (2016-2018) remained higher at 16.0% (13.0%-19.1%; *P* < 0.001) (Table 2).

**Table 2.** Percentage of patients with dementia receiving antipsychotic medications, adjusted for age, sex, and comorbidities, stratified by race and LIS-dual eligibility.

	All^a^		White		Racial and ethnic minority status		LIS-dual		Non-LIS-dual	
Estimated percentage of patients receiving antipsychotics										
2010-2012	9.4%		10.9%		7.7%		9.7%		9.0%	
2013-2015	15.8%		19.3%		10.5%		15.6%		16.3%	
2016-2018	16.0%		17.5%		13.8%		17.4%		14.2%	

Multivariable model										
	Estimate	*P*-value	Estimate	*P*-value	Estimate	*P*-value	Estimate	*P*-value	Estimate	*P*-value
Time										
2010-2012	Ref	—	Ref	—	Ref	—	Ref	—	Ref	—
2013-2015	0.06	<0.001	0.08	<0.001	0.03	0.10	0.06	<0.001	0.07	<0.01
2016-2018	0.07	<0.001	0.07	0.01	0.06	<0.01	0.08	<0.001	0.06	0.10
Age										
65-74	Ref	—	Ref	—	Ref	—	Ref	—	Ref	—
75-84	0.02	0.46	0.04	0.32	−0.02	0.51	0.03	0.31	0.001	0.98
85+	0.06	0.04	0.06	0.13	0.04	0.29	0.04	0.18	0.07	0.12
Sex										
Female	Ref	—	Ref	—	Ref	—	Ref	—	Ref	—
Male	−0.03	0.21	0.01	0.77	−0.09	<0.01	−0.02	0.52	−0.03	0.24
MWI	0.003	<0.01	0.006	<0.001	0.0003	0.85	0.002	0.81	0.002	0.08

^a^Results in the main multivariable model for “all prescriptions” remained significant after applying the Bonferroni correction, with the corrected significance threshold of *P* < 0.017. To interpret our model estimates, we can say that the absolute chance of being prescribed an antipsychotic in the publication period (2013-2015) compared to the reference period (2010-2012) is 6% higher. Similarly, for every 1 unit increase in the MWI, the absolute chance of being prescribed an antipsychotic increases by 0.3%. Abbreviations: LIS-dual, qualifying for either a part D premium low-income subsidy (LIS) or being dual-eligible for both Medicare and Medicaid; MWI, multimorbidity weighted index.

Trends for all prescriptions remained similar after stratifying by White vs racial and ethnic minority status, and LIS-dual vs non-LIS-dual (Table 2). Compared with White patients, racial and ethnic minority status patients were less likely to receive antipsychotics across all timeframes (Table 2). Increases were also seen for potentially low-value prescriptions (3.2%; 6.3%, *P* < 0.01; 5.5%, *P* = 0.06) and potentially indicated prescriptions (6.9%; 9.7%, *P* = 0.02; 10.3%, *P* = 0.02) when comparing pre- vs publication periods, and pre- vs post-publication periods, respectively (Tables S4 and S5). There was no significant increase between the publication and post-publication periods for all (*P* = 0.88), potentially low-value (*P* = 0.52), and potentially indicated (*P* = 0.65) prescriptions.

## Discussion

In this nationally representative analysis of community-dwelling older Americans with dementia, antipsychotic prescriptions increased between the periods before and after the initial publication of CW recommendations and held steady in the subsequent post-publication period.

Our study is among the first to document a rise and plateau in low-value antipsychotic medications, which carry an FDA black box warning for heightened mortality. Our estimated prevalence of overall prescribing is lower than others, which is likely related to different methods of identifying patients with dementia.^12^ Maust et al. estimated 22% of Medicare beneficiaries with dementia were prescribed antipsychotics in 2015-2016, nearly 1.5 times our estimated prevalence of 16% (Table 2). Our results complement the findings of Coe et al.,^7^ who excluded patients with 3 indicated diagnoses (schizophrenia, Huntington's disease, Tourette's syndrome) and noted a slight decrease in low-value antipsychotic prescribing from approximately 11% (2010) to 9% (2018). In our model of potentially low-value prescriptions, which excluded a more comprehensive list of indicated diagnoses (we also excluded bipolar disorder, severe depression, and hospice), our estimates remained lower (3%, 6%, 5%; Table S4). Taking both results from our study and Coe et al., we have more evidence that antipsychotic prescribing has not continued to rise over this time.

The variation in prescribing estimates likely arises from different methods of identifying dementia. Maust and Coe applied a claims-based algorithm to identify dementia, while we took advantage of in-depth cognitive testing available through the HRS. Using ICD codes alone may only capture the most severe presentations of symptomatic dementia and miss subclinical cases.^13^ ICD codes are typically more specific while survey-based methods such as ours may be more sensitive.^13-16^ We likely captured beneficiaries with less severe cases of dementia who received fewer medications. When comparing claims-based methods and survey-based cognitive tests, the concordance in dementia prevalence could be as low as 7%.^13,17^

Some of the increase we observed in overall antipsychotic prescribing could be from an increasing coded prevalence of schizophrenia among patients with dementia (Table S6), a phenomena that has undergone scrutiny by Medicare auditing among nursing homes.^3^ When Shireman et al.^18^ attempted to dissect this phenomena among nursing homes residents, they noted a slight uptick in the incidence of potentially indicated diagnoses (from 2.2% to 2.8% between 2009 and 2018), suggesting the issue was less significant than suggested in the lay press.^3^ To our knowledge, a sister study scrutinizing the true incidence of potentially indicated diagnoses among community-dwelling dementia patients has not yet been performed and is worth exploration.

More broadly, our findings are consistent with our prior work on a wider set of phamaceutical low-value care measures^19^ and other reports on the modest effect of CW related interventions toward reducing low-value care, especially if the intervention is single-pronged and solely educational. A 2021 systematic review demonstrated that multicomponent CW interventions, however, can be effective at changing practice patterns.^20^ Multi-faceted, clinician-targeted interventions, such as novel care models, electronic clinical decision support tools, prescribing benchmarking, and financial incentives, are needed to improve clinician behavior and curb antipsychotic use in this vulnerable population. Importantly, a recent systematic review noted the lack of high-quality evidence for successful discontinuation of antipsychotics in older patients with dementia.^21^ The innovative CMS model Guiding an Improved Dementia Experience launched in 2024 is one of the first to provide caregiver respite and reimburse care coordination.^22^ Given the disparities we noted and the difficulties associated with managing behavioral disturbance at home, future work must examine whether this new model helps to equitably reduce low-value antipsychotic prescribing. Warning clinicians that their antipsychotic prescribing patterns are higher than their peers holds the potential to change prescribing behavior in a sustained way.^23^ We have previously designed and implemented a pragmatic randomized-controlled trial testing a behavioral economics-inspired electronic clinical decision support tool, which reminds clinicians of the hazards of antipsychotic prescribing.^24^ Value-based payment models show promise in decreasing low-value care more broadly,^25^ however they typically exclude part D spending and therefore do not address outpatient antipsychotic prescribing. These models should set clear prescribing benchmarks and incentivize improved prescribing while monitoring for unintended consequences.

###  

#### Strengths and limitations

Given the limitations of claims data, we cannot exclude the possibility of misclassifying prescriptions as low-value when they were potentially indicated (eg, violent agitation) and vice versa. Moreover, the data available to us at the time of this analysis were limited to 2018, thus we are limited in our ability to extrapolate our findings to more current prescribing patterns, particularly with regard to the COVID-19 pandemic, in which numerous challenges related to lockdowns, stay-at-home-requirements, and lack of access to non-pharmacologic interventions contributed to an increase in antipsychotic prescribing for patients with dementia.^26^ Further, results may not generalize to Medicare Advantage beneficiaries who were not included in this study and now represent nearly half of all Medicare beneficiaries.^27^ Compared to Medicare fee-for-service beneficiaries, Medicare Advantage beneficiaries are more likely to be Black or Hispanic, dual-eligible, and reside in urban areas,^27^ and may receive less low-value care due to Medicare Advantage's inherent managed care and utilization control mechanisms.^28^ In an effort to ensure adequate claims history for accurate classification of low-value care, we restricted to beneficiaries with continuous coverage for 36 months. Thus results may not generalize to Medicare beneficiaries with disruptions in coverage. Finally, it is unknown whether prescribing rates would have increased even further than observed in the absence of the CW campaign. Our observational study cannot fully measure the impact of the CW campaign or other confounding factors that may have influenced prescribing over time.

## Conclusion

Antipsychotic prescriptions among community-dwelling older Americans with dementia did not decline after the publication of multiple Choosing Wisely society recommendations, suggesting that stronger interventions are needed to curb overprescribing for this vulnerable group.

## Contribution statement

J.N.M., C.S., C.L.D., and N.H. were responsible for the conception and design of the study, and acquisition of the data. A.W.Y., M.L., and C.-H.T. contributed to the analysis of the data. A.W.Y. and J.N.M. were responsible for drafting the article. All authors contributed to the interpretation of the data, critically revising the article for important intellectual content, and final approval of the manuscript. Sponsors did not have a role in the acquisition of data, design of the study, interpretation of data, or preparation of the manuscript.

## Supplementary Material

qxaf021_Supplementary_Data
