# Supplementary material for: Trends in antipsychotic prescribing among community-dwelling older adults with dementia, 2010-2018
Source: Health Aff Sch. 2025 Feb 26;3(2):qxaf021. doi: 10.1093/haschl/qxaf021 (PMC11878382; doi:10.1093/haschl/qxaf021)
Supplement: qxaf021_Supplementary_Data [file qxaf021_supplementary_data.zip › Supplement HA Scholar_02.08.2025.docx]

**SUPPLEMENT**

*Cohort*

We created our cohort using previously described methods, using the Health and Retirement Study (HRS) to identify Medicare beneficiaries 65 years and older with Medicare fee-for-service, living with dementia between 2008 and 2018. The HRS is a nationally representative, longitudinal, biennial survey administered to adults 50 year and older and collects information on wealth, employment, family, health, and cognition.^1^ Our dataset was not linked to claims for patients covered under Medicare Advantage.

Beneficiaries needed to have continuous coverage of Medicare Part A, B, and Part D prescription drug coverage for 24 months prior to the year of study and for the entire 12 months of the measurement year of interest. The 24-month lookback period ensured an adequate amount of time for determining one’s clinical history. Since we looked for antipsychotic use anytime during the measurement year of interest, we required 12 months of coverage in this year to increase the validity of our results. This ensured everyone contributed the same amount of time “at-risk” to receive a prescription. Thus, we only start reporting data from 2010 because of the required 24-month lookback period. Furthermore, by requiring 24 months of claims history, we were attempting to avoid misclassification errors of omission in measuring low-value care. Generally, the longer the look back period, the more likely the algorithm can detect valid exclusion criteria (e.g., bipolar disorder) for antipsychotic prescriptions.

We excluded beneficiaries who had long term nursing home stays, defined as ≥100 cumulative days in a facility, using SNF claims.^2^ We calculated length of nursing home stays by taking the difference between the “admission” and “discharge” date. The admission date could be from a previous calendar year, as long as the beneficiary was discharged in the given calendar year.

Please see Supplement Figure 1 for a CONSORT diagram representing the unweighted sample of all unique participants between 2010-2018.

Given that the survey is only administered biennially, population weights and survey data during the “off” odd-numbered years were imputed from data obtained during the prior survey year (e.g. 2011 data was imputed from 2010 HRS data).

*Identification of dementia*

Detailed methods for dementia identification have been described elsewhere.^3-6^ In brief, we identified patients with dementia using two different scales, depending on whether the patient could respond on their own (27-point scale), or whether a health care proxy responded on their behalf (11-point scale). When respondents could not respond, their health care proxy answered on their behalf, which helped mitigate the issue of missing data and non-response bias. We further categorized the severity of dementia as mild, moderate, or severe, based on previously validated methods.^7^ Both scales were based on the telephone interview for cognitive status (TICS) which includes immediate and delayed 10-noun free recall testing, serial 7 subtraction testing, and backwards count from 20. The following thresholds were used to assess dementia severity, for the 27-point and 11-point scales respectively: (1) mild dementia: 5-6 and 6; (2) moderate dementia: 3-4 and 7; and (3) severe dementia: 0-2 and 8-11. These cognitive measures have been well validated in prior studies.^3-5,7^ It is theoretically possible for some patients to be diagnosed with dementia during one of the survey years and then to no longer qualify as having dementia in a subsequent survey year, and vice versa.

*Demographics and co-morbidities*

We reported basic demographic information, including sex, race, geographic location, education, and presence of a caregiver. Race was classified using HRS data (Non-Hispanic White, Non-Hispanic Black, Hispanic, Other) given that responses were self-reported.

We assessed comorbidities using the multimorbidity weighted index (MWI). The MWI was developed by Wei and colleagues,^8^ and has been validated using HRS data and mapped to ICD9 codes. Briefly, the MWI weights 81 chronic diseases and conditions by their impact on physical health-related quality of life, has strong associations with mortality, and stronger associations with future physical functioning than previous illness burden algorithms such as the Charlson Index and Elixhauser Score.^8^

*Socioeconomic status*

We used two measures for socioeconomic disadvantage per Medicare: (1) dual-eligibility status and (2) eligibility for a Part D premium low-income subsidy (LIS) as proxies for lower socioeconomic status. Dual eligibility status was defined using claims data as being enrolled in Medicaid for ≥1 month during the year of interest. Beneficiaries were considered eligible for the LIS if they were eligible for ≥1 months of the year of interest.

*Antipsychotic Prescribing*

Exposure to antipsychotic prescribing was determined using prescription fills from January 1 to December 31 of each study year. We compiled a list of antipsychotic agents and identified NDC codes for each drug (Supplement Table 1). NDC codes are available upon request and were last updated December 2022. We searched the Part D file for patients who received ≥1 day supply of any of the listed antipsychotic agents.

We used specific diagnoses to categorize antipsychotic prescribing into four outcomes: (1) All prescriptions (2) Potentially indicated; (3) Possibly Low-Value; (4) Probably Low-Value (Supplement Table 2). Diagnoses for each category were compiled from a review of prior literature and were also reviewed with clinicians on the research team.^2,9,10^ The three categories of “potentially indicated,” “possibly low-value”, and “probably low-value” were all mutually exclusive. We identified ICD-9 and ICD-10 codes for all diagnoses, and searched inpatient, outpatient, and carrier data files for the presence of these ICD codes in the 24 months prior to the year of study and anytime within the study year. If diagnoses from multiple categories were present, we assigned the most “conservative category.” For example, if both schizophrenia (potentially indicated) and mild depression (probable low-value) were present, then the prescription was categorized as “potentially indicated.”

Enrollment in hospice was identified using the HRS Hospice file. We searched the hospice file in the 24 months prior the study year and during the study year.

We identified depression using both ICD codes and HRS data by using the short-form version of the Composite International Diagnostic Interview (CIDI-SF) module for major depression. The CIDI-SF is derived from the *Diagnostic and Statistical Manual of Mental Disorders* criteria and has been validated with clinical psychiatric interviews.^11^ For each study year, the CIDI-SF score was determined either using the survey from the measurement year of interest, or data from the prior survey year. We followed the screening pathway previously described by Dang et al (see Dang Supplemental Figure 1).^11^ Patients could either be screened into completing the CIDI-SF through either the “Dysphoria” pathway or the “Anhedonia” pathway. Patients who complete the CIDI-SF through the “Dysphoria” pathway could score a maximum of 7, while patients who complete the CIDI-SF through the “Anhedonia” pathway could score a maximum of 6. The following CIDI-SF score thresholds were used for determining the severity of depression: (1) Mild or moderate depression: score 3-4; (2) Severe depression: score ≥5.

*Statistical Analysis*

For each year, we calculated the annual prescribing rate per 1000 beneficiaries and then calculated the average prescribing rate across the three time periods of interest: 2010-2012 (pre-publication of recommendations) vs 2013-2015 (publication of recommendations by American Geriatrics Society, the Society for Post-Acute and Long-Term Care Medicine, the American Psychiatric Association), and 2010-2012 vs 2016-2018 (after publication of recommendations). For each prescribing outcome as above, we constructed univariate models with antipsychotic prescribing as the outcome and the three time periods as the independent variable. We used a multivariable mixed regression model accounting for within subject effects, with the same three time periods as the independent variable, adjusting for age, sex, and comorbidities using the multimorbidity weighted index (MWI) as described above. Using the multivariable model, we estimated the probability of patients being prescribed antipsychotics for each time period. For the main outcome of “all prescriptions”, the Bonferroni correction was applied to control the type I error rate at the 5% level, to address multiple testing between time periods. We accounted for survey stratification and clustering and applied survey weights for national representativeness and response rate.

**Supplement Figure 1.** CONSORT diagram for unique HRS-Medicare survey participants, 2010-2018

**
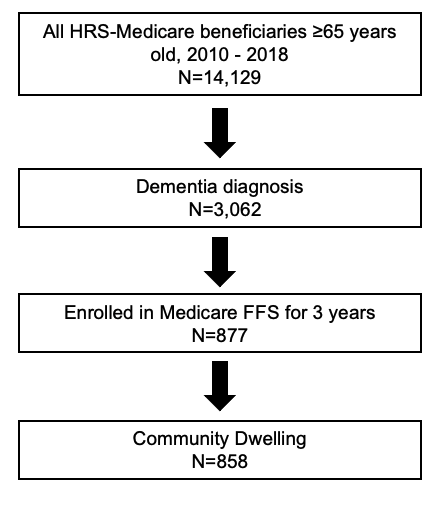
**

**Supplement Figure 2.** Unadjusted rate of antipsychotic prescribing per 1000 patients with dementia as related to the release of Choosing Widely recommendations.


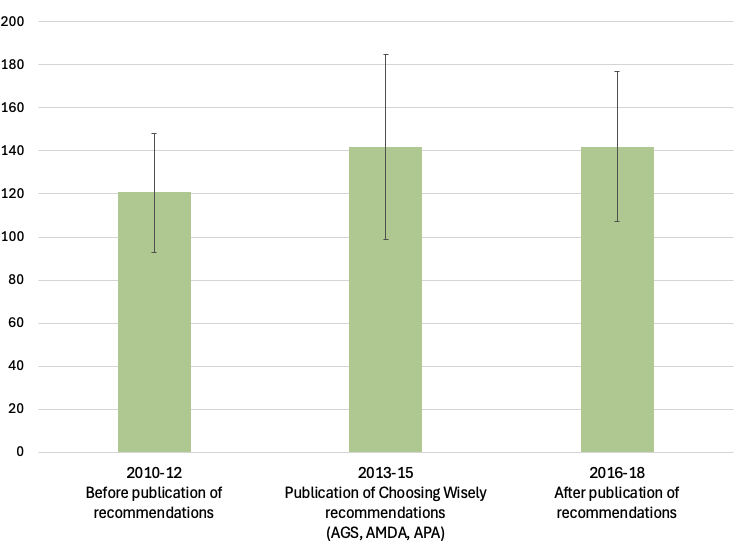


Figure Legend:

AGS = American Geriatrics Society, AMDA = Post-Acute and Long-Term Care Medicine, APA = American Psychiatric Association

**SUPPLEMENT TABLE 1.** List of antipsychotic agents

| Antipsychotics | Aripiprazole  Asenapine  Brexpiprazole  Cariprazine  Chlorpromazine  Chlorprothixene  Clozapine  Fluphenazine  Haloperidol  Iloperidone  Loxapine  Lumateperone  Lurasidone  Mesoridazine besylate  Methotrimeprazine  Molindone  Olanzapine  Paliperidone  Perphenazine  Pimavanserin  Pimozide  Promazine  Quetiapine  Risperidone  Thioridazine  Thiothixene  Trifluoperazine  Triflupromazine  Ziprasidone |
| --- | --- |

NDC codes are available upon request.

**SUPPLEMENT TABLE 2.** Clinical indications and diagnoses for levels of appropriate prescribing of antipsychotics

| Clinical Appropriateness | Diagnoses |
| --- | --- |
| Potentially Indicated | Hospice (HRS Hospice file)  Schizophrenia  Bipolar disorder  Tourette Syndrome  Huntington’s Disease  Severe depression (CIDI score ≥5; or ICD codes) |
| Possibly Low Value | Mild or moderate depression (CIDI score 3-4, or ICD codes) |
| Probably Low Value | All other diagnoses |

CIDI = Composite International Diagnostic Interview score

**SUPPLEMENT TABLE 3.** STROBE Checklist for cohort studies

|  | Item No | Recommendation |
| --- | --- | --- |
| **Title and abstract** | 1 | (*a*) The study’s design is indicated to be a retrospective-cohort analysis in the abstract. |
|  |  | (*b*) An abstract summarizes what was done and what was found. |
| Introduction | | |
| Background/rationale | 2 | The rationale for the reported investigation is in the introduction. |
| Objectives | 3 | Specific objectives of this investigation are in the introduction. |
| Methods | | |
| Study design | 4 | Key elements of study design are presented in the methods section, and in the Online Supplemental Appendix. |
| Setting | 5 | The data from this study comes from the Health and Retirement Survey described in the manuscript body, and in the Online Supplemental Appendix. |
| Participants | 6 | The eligibility criteria are described in the Methods. |
| Variables | 7 | We have clearly defined our primary outcome, which is percentage of patients prescribed any antipsychotic in each year. This is a descriptive study that does not make causal inference; therefore, we do not need to address confounders. |
| Data sources/ measurement | 8 | For each variable of interest, the source of the data and details of method assessment are detailed in Methods and the Online Supplemental Appendix. |
| Bias | 9 | Efforts to mitigate non-response bias and missing data in the Health and Retirement Survey (HRS) are described in the Online Supplemental Appendix. |
| Study size | 10 | A description of how we arrived at the study size is contained in the Online Supplemental Appendix. |
| Quantitative variables | 11 | An explanation of how quantitative variables were used in the analyses is in the Online Supplemental Appendix. |
| Statistical methods | 12 | (*a*) All statistical methods are described in the Methods section and the Online Supplemental Appendix. |
|  |  | (*b*) We stratify our outcomes by race/ethnicity and socioeconomic status. An explanation is in the Methods. |
|  |  | (*c*) An explanation of how the missing data were addressed is in the Online Supplemental Appendix. |
|  |  | (*d*) N/A |
|  |  | (*e*) N/A |
| Results | | |
| Participants | 13* | (a) The number of individuals eligible for the study is reported in the Results. |
|  |  | (b) We constructed a CONSORT diagram (Supplement Figure 1) |
|  |  | (c) We constructed a CONSORT diagram (Supplement Figure 1) |
| Descriptive data | 14* | (a) We provide descriptive characteristics of the study participants, including average age, gender, and race/ethnicity in the Results and Table 1. |
| Outcome data | 15* | Our primary outcome is the percentage of patients prescribed any antipsychotic in each year, Supplement Figure 2 and Table 2. |
| Main results | 16 | (*a*) Unadjusted estimates with 95% confidence intervals are included in the Results and Supplement Figure 2. |
|  |  | (*b*) We provide category boundaries in our dementia staging definitions, Online Supplemental Appendix. |
|  |  | (*c*) N/A. |
| Other analyses | 17 | Other analyses are reported in the Online Supplemental Tables 4-6. |
| Discussion | | |
| Key results | 18 | Key results are summarized in the Discussion, paragraphs 1-2. |
| Limitations | 19 | The limitations of the study are outlined in the Discussion. |
| Interpretation | 20 | Our overall interpretation of the results is in the Discussion, paragraphs 1-2. |
| Generalizability | 21 | The generalizability of the study is discussed in the entire discussion section, including study limitations. |
| Other information | | |
| Funding | 22 | We have identified and acknowledged the funders of this study in the Acknowledgements section. |

**SUPPLMEMENT TABLE 4.** Percentage of patients receiving potentially low-value antipsychotics, adjusted for age, sex, and co-morbidities, stratified by race and LIS-Dual eligibility

|  | **All** | | **White** | | **Non-White** | | **LIS-Dual** | | **Non LIS-Dual** | |
| --- | --- | --- | --- | --- | --- | --- | --- | --- | --- | --- |
| Estimated percentage of patients receiving potentially low value antipsychotics | | | | | | | | | | |
| 2010-12 | 3.2% |  | 3.8% |  | 2.6% |  | 4.1% |  | 1.1% |  |
| 2013-15 | 6.3% |  | 8.7% |  | 2.8% |  | 4.5% |  | 9.2% |  |
| 2016-18 | 5.5% |  | 5.1% |  | 6.0% |  | 4.6% |  | 6.4% |  |
| Multivariable Model | | | | | | | | | | |
|  | Estimate | P Value | Estimate | P Value | Estimate | P Value | Estimate | P Value | Estimate | P Value |
| Time | | | | | | | | | | |
| 2010-12 | Ref | – | Ref | – | Ref | – | Ref | – | Ref | – |
| 2013-15 | 0.03 | <.01 | 0.05 | <.01 | 0.002 | 0.91 | 0.042 | 0.78 | 0.08 | <.001 |
| 2016-18 | 0.02 | 0.06 | 0.01 | 0.50 | 0.03 | 0.02 | 0.006 | 0.72 | 0.05 | 0.02 |
|  |  |  |  |  |  |  |  |  |  |  |
| Age |  |  |  |  |  |  |  |  |  |  |
| 65-74 | Ref | – | Ref | – | Ref | – | Ref | – | Ref | – |
| 75-84 | 0.01 | 0.47 | 0.02 | 0.32 | -0.009 | 0.64 | 0.003 | 0.87 | 0.03 | 0.38 |
| 85+ | 0.03 | 0.09 | 0.05 | 0.08 | -0.002 | 0.94 | 0.017 | 0.41 | 0.04 | 0.20 |
|  |  |  |  |  |  |  |  |  |  |  |
| Sex |  |  |  |  |  |  |  |  |  |  |
| Female | Ref | – | Ref | – | Ref | – | Ref | – | Ref | – |
| Male | -0.01 | 0.69 | 0.02 | 0.36 | -0.05 | 0.01 | 0.008 | 0.47 | -0.02 | 0.29 |
|  |  |  |  |  |  |  |  |  |  |  |
| MWI | -0.001 | 0.55 | <.001 | 0.83 | -0.002 | 0.07 | -0.001 | 0.28 | <.001 | 0.56 |

“Potentially low-value” prescribing includes both possibly and probably low-value prescriptions.

**SUPPLEMENT TABLE 5.** Percentage of patients receiving potentially indicated antipsychotics, adjusted for age, sex, and co-morbidities, stratified by race and LIS-Dual eligibility

|  | **All** | | **White** | | **Non-White** | | **LIS-Dual** | | **Non LIS-Dual** | |
| --- | --- | --- | --- | --- | --- | --- | --- | --- | --- | --- |
| Estimated percentage of patients receiving potentially indicated antipsychotics | | | | | | | | | | |
| 2010-12 | 6.9% |  | 7.6% |  | 5.9% |  | 6.6% |  | 8.7% |  |
| 2013-15 | 9.7% |  | 10.4% |  | 8.1% |  | 11.5% |  | 7.4% |  |
| 2016-18 | 10.3% |  | 12.3% |  | 7.6% |  | 12.6% |  | 7.8% |  |
| Multivariable Model | | | | | | | | | | |
|  | Estimate | P Value | Estimate | P Value | Estimate | P Value | Estimate | P Value | Estimate | P Value |
| Time | | | | | | | | | | |
| 2010-12 | Ref | – | Ref | – | Ref | – | Ref | – | Ref | – |
| 2013-15 | 0.03 | 0.02 | 0.03 | 0.12 | 0.02 | 0.12 | 0.05 | <.001 | -0.01 | 0.56 |
| 2016-18 | 0.03 | 0.02 | 0.05 | 0.03 | 0.02 | 0.32 | 0.06 | <.001 | -0.009 | 0.71 |
|  |  |  |  |  |  |  |  |  |  |  |
| Age |  |  |  |  |  |  |  |  |  |  |
| 65-74 | Ref | – | Ref | – | Ref | – | Ref | – | Ref | – |
| 75-84 | 0.01 | 0.52 | 0.01 | 0.58 | 0.005 | 0.83 | 0.04 | 0.14 | -0.02 | 0.54 |
| 85+ | 0.03 | 0.12 | 0.02 | 0.53 | 0.04 | 0.11 | 0.03 | 0.21 | 0.04 | 0.27 |
|  |  |  |  |  |  |  |  |  |  |  |
| Sex |  |  |  |  |  |  |  |  |  |  |
| Female | Ref | – | Ref | – | Ref | – | Ref | – | Ref | – |
| Male | -0.02 | 0.23 | -0.01 | 0.70 | -0.04 | 0.10 | -0.02 | 0.21 | -0.01 | 0.67 |
|  |  |  |  |  |  |  |  |  |  |  |
| MWI | 0.004 | <.001 | 0.004 | <.001 | 0.003 | 0.02 | 0.004 | <.001 | 0.003 | 0.09 |

**SUPPLEMENT TABLE 6**. HRS-Medicare beneficiaries with schizophrenia, stratified by presence of dementia

| **Beneficiaries with schizophrenia** | **2010** | **2011** | **2012** | **2013** | **2014** | **2015** | **2016** | **2017** | **2018** |
| --- | --- | --- | --- | --- | --- | --- | --- | --- | --- |
| Without dementia (%) | 82.35 | 78.71 | 81.87 | 89.87 | 60.47 | 69.67 | 65.56 | 66.47 | 57.15 |
| With dementia (%) | 17.65 | 21.29 | 18.13 | 10.13 | 39.53 | 30.33 | 34.44 | 33.53 | 42.85 |
| Prescribed antipsychotic (%)^a^ | 60.14 | 61.23 | 26.79 | 82.26 | 86.78 | 51.81 | 100.00 | 80.89 | 86.25 |

^a^ Of patients with schizophrenia and dementia, the percentage who were prescribed an antipsychotic

**SUPPLEMENT REFERENCES**

1. Sonnega A, Faul JD, Ofstedal MB, Langa KM, Phillips JW, Weir DR. Cohort Profile: the Health and Retirement Study (HRS). *Int J Epidemiol*. Apr 2014;43(2):576-85. doi:10.1093/ije/dyu067

2. Centers for Medicare & Medicaid Services. *Antipsychotic Use in Part D Enrollees with Dementia*. Medicare Drug Benefit and C & D Data Group: Division of Clinical and Operational Performance; 2015. November 16, 2015. Accessed January 20, 2025. <https://www.cms.gov/Medicare/Prescription-Drug-Coverage/PrescriptionDrugCovGenIn/Downloads/Antipsychotic-Use-in-Part-D-Enrollees-with-Dementia-v12092015.pdf>

3. Langa KM, Plassman BL, Wallace RB, et al. The Aging, Demographics, and Memory Study: study design and methods. *Neuroepidemiology*. 2005;25(4):181-91. doi:10.1159/000087448

4. Langa KM, Larson EB, Crimmins EM, et al. A Comparison of the Prevalence of Dementia in the United States in 2000 and 2012. *JAMA Intern Med*. Jan 1 2017;177(1):51-58. doi:10.1001/jamainternmed.2016.6807

5. Crimmins EM, Kim JK, Langa KM, Weir DR. Assessment of cognition using surveys and neuropsychological assessment: the Health and Retirement Study and the Aging, Demographics, and Memory Study. *J Gerontol B Psychol Sci Soc Sci*. Jul 2011;66 Suppl 1(Suppl 1):i162-71. doi:10.1093/geronb/gbr048

6. Mafi JN, Leng M, Arbanas JC, et al. Estimated Annual Spending on Aducanumab in the US Medicare Program. *JAMA Health Forum*. 2022;3(1):e214495-e214495. doi:10.1001/jamahealthforum.2021.4495

7. Langa KM, Chernew ME, Kabeto MU, et al. National estimates of the quantity and cost of informal caregiving for the elderly with dementia. *J Gen Intern Med*. Nov 2001;16(11):770-8. doi:10.1111/j.1525-1497.2001.10123.x

8. Wei MY, Ratz D, Mukamal KJ. Multimorbidity in Medicare Beneficiaries: Performance of an ICD-Coded Multimorbidity-Weighted Index. *J Am Geriatr Soc*. May 2020;68(5):999-1006. doi:10.1111/jgs.16310

9. U.S. Government Accountability Office. *Antipsychotic Drug Use: HHS Has Initiatives to Reduce Use among Older Adults in Nursing Homes, but Should Expand Efforts to Other Settings*. 2015. Accessed December 21, 2022. <https://www.gao.gov/products/gao-15-211>

10. Gerlach LB, Kales HC, Kim HM, et al. Trends in Antipsychotic and Mood Stabilizer Prescribing in Long-Term Care in the U.S.: 2011-2014. *J Am Med Dir Assoc*. Nov 2020;21(11):1629-1635.e8. doi:10.1016/j.jamda.2020.05.039

11. Dang L, Dong L, Mezuk B. Shades of Blue and Gray: A Comparison of the Center for Epidemiologic Studies Depression Scale and the Composite International Diagnostic Interview for Assessment of Depression Syndrome in Later Life. *Gerontologist*. May 15 2020;60(4):e242-e253. doi:10.1093/geront/gnz044
