# Supplementary material for: Trends in antipsychotic prescribing among community-dwelling older adults with dementia, 2010-2018
Source: Health Aff Sch. 2025 Feb 26;3(2):qxaf021. doi: 10.1093/haschl/qxaf021 (PMC11878382; doi:10.1093/haschl/qxaf021)
Supplement: qxaf021_Supplementary_Data [file qxaf021_supplementary_data.zip › Supplement Figure 1_CONSORT Diagram.pdf]

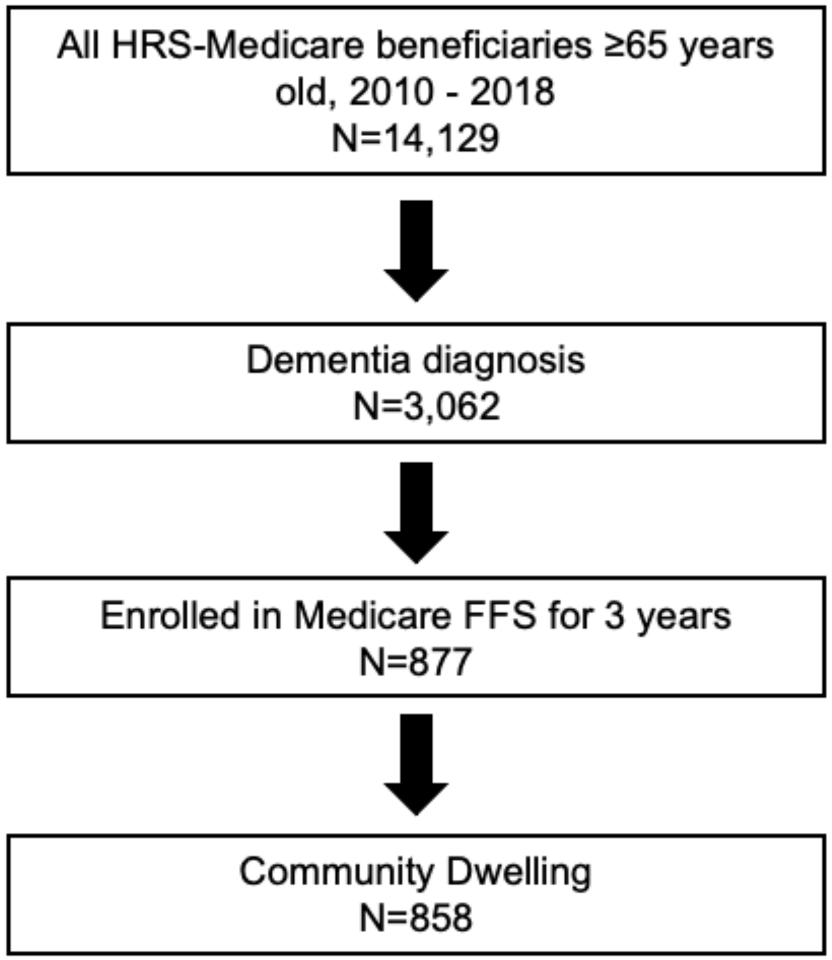
All HRS-Medicare beneficiaries ≥65 years old, 2010 - 2018
N=14,129
Dementia diagnosis
N=3,062
Enrolled in Medicare FFS for 3 years
N=877
Community Dwelling
N=858
