# Supplementary figures and images for: Trends in antipsychotic prescribing among community-dwelling older adults with dementia, 2010-2018
Source: Health Aff Sch. 2025 Feb 26;3(2):qxaf021. doi: 10.1093/haschl/qxaf021 (PMC11878382; doi:10.1093/haschl/qxaf021)

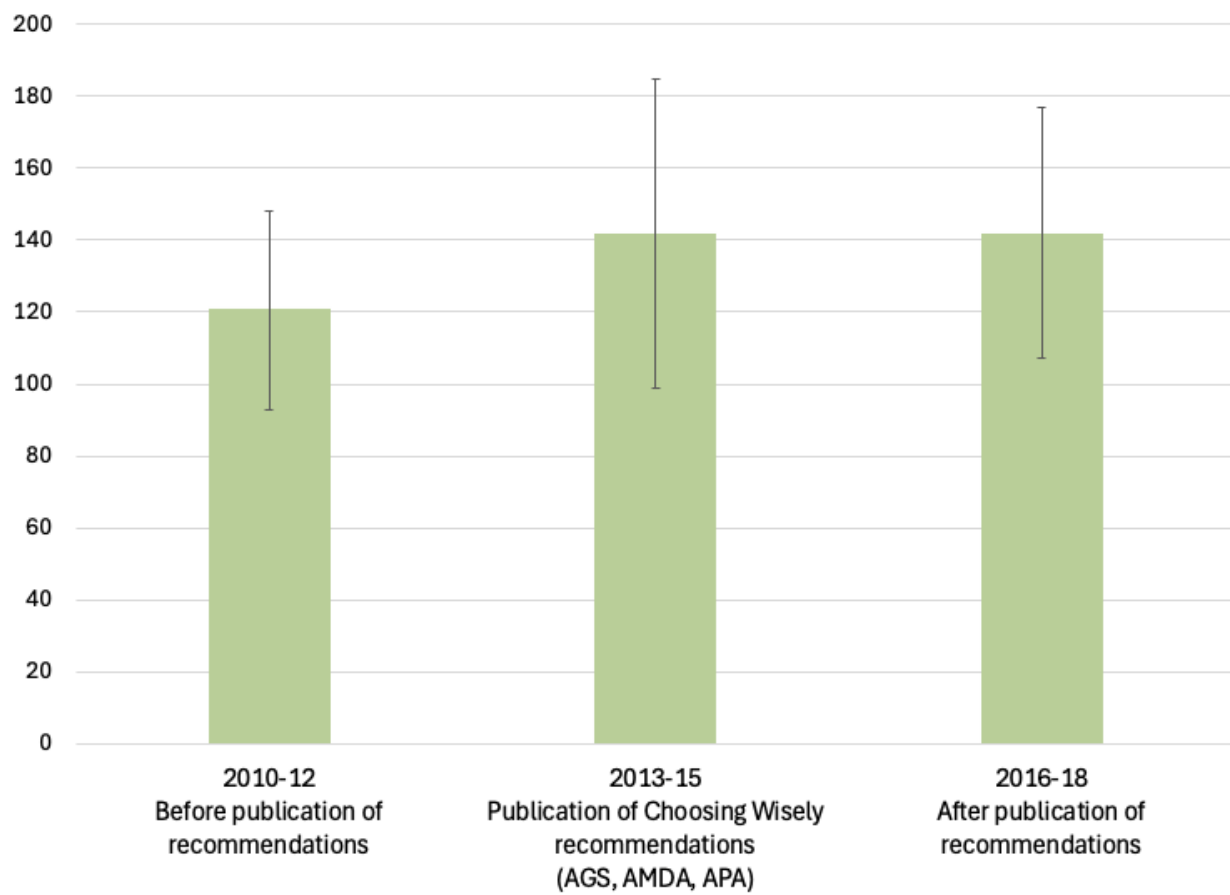

Supplement: qxaf021_Supplementary_Data [file qxaf021_supplementary_data.zip › Supplement Figure 2_Unadjusted prescribing rate.pdf]
